# Toxicological Effects of Phthalate Plasticizers in Zebrafish Models: A Review

**DOI:** 10.3390/molecules31122024

**Published:** 2026-06-09

**Authors:** Shiqiao Wang, Hongming Hou, Fengxian Qin, Chang Sun, Chengyu Lv, Tiezhu Li, Jie Zhang

**Affiliations:** 1 College of Food Science and Engineering, Jilin Agricultural University, Changchun 130118, China; 2 Jilin Provincial Laboratory of Crop Germplasm Resources, Agronomy College, Jilin Agricultural University, Changchun 130118, China; 3 Institute of Agro-Food Technology, Jilin Academy of Agricultural Sciences (Northeast Agricultural Research Center of China), Changchun 130033, China; 4 College of Food Science and Engineering, Jilin University, Changchun 130062, China

**Keywords:** phthalic acid esters (PAEs), zebrafish, developmental toxicity, risk assessment

## Abstract

Phthalic acid esters (PAEs), ubiquitous plasticizers and recognized endocrine-disrupting chemicals, pose a protracted threat to aquatic ecosystems and biodiversity. However, current ecotoxicological assessments often focus on isolated chemicals at exceedingly high laboratory doses, failing to reflect true environmental risks. This review systematically evaluates and compares the multisystemic toxicological effects of six priority PAEs (DEHP, DBP, BBP, DNOP, DEP, and DMP) using the zebrafish biological model. The synthesized evidence reveals a distinct structure–activity relationship, where long-chain and highly hydrophobic congeners exhibit substantially higher toxicity than their short-chain counterparts. Exposure to these PAEs induces severe developmental, cardiovascular, neurobehavioral, and reproductive anomalies. Specifically, DBP and BBP display the most potent cardiotoxic and neurotoxic effects, while DEHP and DBP drive profound reproductive decline and endocrine disruption at concentrations as low as 0.5–20 μg/L. Crucially, comparative environmental relevance assessments indicate that real-world PAE concentrations in industrial hotspots frequently meet or exceed these laboratory-derived lowest observed effect concentrations. These findings underscore the severe ecological risks posed by PAE contamination and position the zebrafish as a vital biological sentinel. Future ecotoxicological evaluations must prioritize chronic, low-dose mixture exposures and transgenerational toxicity to fully characterize the protracted legacy of these pollutants on zebrafish populations.

## 1. Introduction

Modern society has been aptly termed the “Plastic Age”. Currently, global plastic production has reached approximately 400 million tons annually, a figure projected to double by 2050. It is estimated that nearly 6.3 billion tons of plastic waste have been generated globally, of which roughly 79% has accumulated in landfills and natural environments [1]. Due to their extreme persistence and slow degradation rates, plastics have become ubiquitous environmental pollutants. Microplastic contamination has pervaded almost all ecosystems, encompassing marine, freshwater, terrestrial, and even Antarctic snow environments, with recent evidence confirming their presence in human blood [2,3]. The sheer volume of these materials ensures that aquatic environments serve as the ultimate sink for plastic debris, creating a profound and fundamental environmental challenge. Beyond aquatic toxicity, growing concerns have also been raised regarding the potential contribution of environmental pollutants to chronic metabolic and cardiovascular disorders in humans [4]. Increasing attention has also been directed toward the toxicodynamic and toxicokinetic behavior of environmental contaminants across food chains and aquatic ecosystems [5].

Accompanying this explosive growth in plastic pollution is the continuous release of chemical additives into aquatic ecosystems. Among these, phthalic acid esters (PAEs), the most extensively used plasticizers globally, have been recognized as potent endocrine-disrupting chemicals (EDCs) that pose a protracted threat to ecological integrity and human health [6]. Increasing evidence also suggests that chronic environmental contaminants may induce systemic oxidative stress and inflammatory dysregulation, thereby contributing to broader neuroendocrine and cardiovascular health disturbances [7,8]. Because they are merely physically blended rather than chemically bound to polymer matrices, PAEs leach readily into surrounding water bodies. Given their exceptionally high environmental detection frequencies and significant ecotoxicological risks, six representative PAEs—di-(2-ethylhexyl) phthalate (DEHP), di-n-butyl phthalate (DBP), butyl benzyl phthalate (BBP), di-n-octyl phthalate (DNOP), diethyl phthalate (DEP), and dimethyl phthalate (DMP)—have been listed as priority pollutants by the United States Environmental Protection Agency (US EPA) and other regulatory bodies [9,10,11]. Their chronic accumulation in various water bodies urgently necessitates precise ecotoxicological evaluations to map their descriptive toxicity profiles.

To accurately assess the toxic effects of these priority PAEs on aquatic life, it is essential to select an appropriate in vivo biological model. The zebrafish (*Danio rerio*) has become a widely accepted vertebrate model for aquatic toxicity testing and chemical screening due to its suitability for evaluating developmental, reproductive, cardiovascular, and neurobehavioral toxicity [12,13]. Compared with traditional mammalian models, zebrafish offer several distinct advantages, including transparent embryos that allow direct, non-invasive observation of macroscopic developmental defects and morphological alterations [14,15]. Furthermore, their high fecundity, rapid development, and compatibility with high-throughput phenotypic screening make them an ideal model for systematically comparing the observable toxicological endpoints of different environmental contaminants. Environmental pollutants capable of disrupting cardiovascular and neuroendocrine homeostasis have attracted increasing attention due to their potential long-term implications for organismal health and ecological stability [16]. Despite a growing number of studies using zebrafish to evaluate PAE toxicity, the existing literature focused on the isolated effects of single chemicals, frequently under acute exposures and at very high laboratory doses. Increasing attention has also been directed toward the long-term effects of environmental stressors on neuroendocrine regulation and behavioral health, further emphasizing the importance of evaluating environmentally relevant toxicants [17]. Consequently, a systematic comparative analysis of the diverse phenotypic toxicities of these six priority PAEs remains urgently needed. Therefore, this review aims to systematically summarize and compare the specific, observable toxicological endpoints induced by these six PAEs in the zebrafish model, including gross developmental malformations, cardiovascular abnormalities, neurological behavioral shifts, and reproductive decline. We comparatively synthesize and discuss the reported toxicological effects observed under laboratory exposure conditions and environmentally relevant concentrations to better evaluate their ecological significance. This comparative narrative review integrates toxicological evidence across multiple PAE congeners and physiological systems in zebrafish models. Accordingly, the central research question addressed is: how do differences in physicochemical properties among representative PAEs influence their multisystem toxicological outcomes and ecological risk relevance in zebrafish models?

To achieve these objectives, the remainder of this review is structured as follows. Section 2 describes the literature search methodology, including the databases consulted, search terms, and inclusion and exclusion criteria. Section 3 summarizes the physicochemical characteristics of the representative PAEs and how these properties dictate their exposure relevance in zebrafish models. Section 4 systematically compares the multisystemic, phenotypic toxicological effects induced by the six priority PAEs, highlighting the most sensitive biological endpoints. Section 5 evaluates the environmental relevance by comparing laboratory-derived toxicity thresholds with real-world exposure risks in aquatic ecosystems. Section 6 acknowledges the limitations of the present review. Finally, Section 7 presents concluding remarks and outlines future perspectives for ecotoxicological assessments.

## 2. Literature Search Methodology

Relevant literature regarding the toxicological effects of PAEs in zebrafish models was systematically searched using the Web of Science, PubMed, Scopus, and Google Scholar databases. The literature search also covered publications available up to March 2026.

The primary search terms included combinations of “phthalic acid esters”, “PAEs”, “DEHP”, “DBP”, “BBP”, “DNOP”, “DEP”, “DMP”, “zebrafish”, “Danio rerio”, “developmental toxicity”, “cardiotoxicity”, “neurotoxicity”, “reproductive toxicity”, and “endocrine disruption”.

Studies were included if they:

(1) Investigated the toxicological effects of representative PAEs using zebrafish models;

(2) Reported observable phenotypic, biochemical, molecular, or behavioral endpoints;

(3) Were published in peer-reviewed journals in English.

Studies were excluded if they:

(1) Lacked original experimental data;

(2) Focused exclusively on non-zebrafish models; or

(3) Did not provide sufficient toxicological outcome information relevant to this review.

Literature screening and data extraction were independently conducted by two authors, and disagreements were resolved through discussion among all authors.

**Figure 1:**
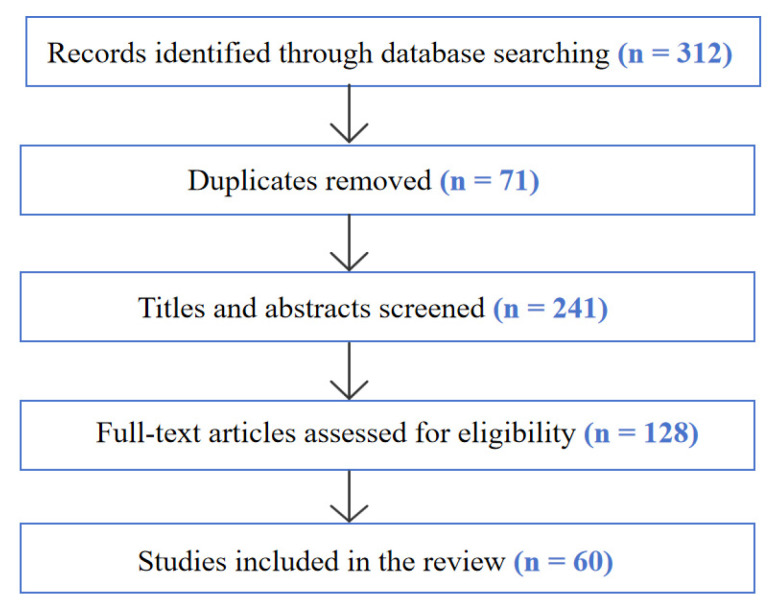
Flow diagram of the literature screening process for the review. The number of records at each stage is indicated in parentheses.

Considering the comparative and narrative nature of this review, a formal meta-analysis was not performed. Instead, emphasis was placed on comparing toxicological endpoints, exposure concentrations, and environmentally relevant toxicity thresholds across studies. Although a formal risk-of-bias scoring system was not applied, studies with insufficient experimental details or unclear exposure conditions were excluded from comparative interpretation whenever possible. The literature screening process is summarized in Figure 1, which illustrates the number of records identified, excluded, and retained at each stage of the selection procedure.

## 3. Physicochemical Characteristics of Representative PAEs and Their Exposure Relevance in Zebrafish Models

In this review, descriptive toxicity refers to observable phenotypic endpoints in zebrafish, including developmental malformations, behavioral abnormalities, and mortality, as opposed to mechanistic toxicity, which involves molecular or cellular pathways underlying these effects. The concept of descriptive toxicity is used here to summarize observable endpoints across studies, providing a comparative overview of compound effects, while mechanistic toxicity is discussed where molecular or biochemical data are available.

PAEs are a class of synthetic organic chemicals [18]. Industrially, the six priority PAEs discussed in this review are incorporated into a vast array of consumer products, including PVC building materials, medical devices, food packaging, and personal care products [19,20]. Crucially, PAEs are not chemically bound to the plastic polymer matrix; they are merely physically blended [21]. Consequently, they are highly susceptible to leaching into the surrounding environment upon exposure to physical wear, temperature changes, or aging [22,23]. Due to this continuous release, aquatic ecosystems—including rivers, lakes, and wastewater effluents—have become the major environmental sink for these contaminants [24].

The physical and chemical properties of these six PAEs, primarily their molecular weight (MW) and water solubility, create distinct differences in their toxicity profiles in zebrafish. Low-molecular-weight PAEs have shorter side chains and relatively higher water solubility [25,26]. In zebrafish exposure models, they are easily dissolved in water and absorbed, often leading to specific acute toxicity and observable morphological endpoints. Conversely, high-molecular-weight PAEs are highly hydrophobic [27]. Their strong lipophilicity allows them to readily accumulate in lipid-rich tissues of the zebrafish, such as the embryonic yolk sac [28,29]. This accumulation often results in different phenotypic alterations, particularly prolonged developmental and reproductive anomalies.

Historically, numerous zebrafish toxicological assays employed extraordinarily high exposure doses to effectively observe acute lethality and severe macroscopic malformations. While these high-dose studies are useful for identifying potential toxic hazards, they often deviate from actual ecological scenarios. Therefore, the subsequent sections will systematically review and compare the specific toxicological endpoints induced by these PAEs, with a special emphasis on the observed phenotypic differences between high laboratory doses and environmentally relevant exposures.

## 4. Multisystemic Toxicological Effects of PAEs in Zebrafish: Phenotypic Characterization and Sensitive Endpoints

**Figure 2:**
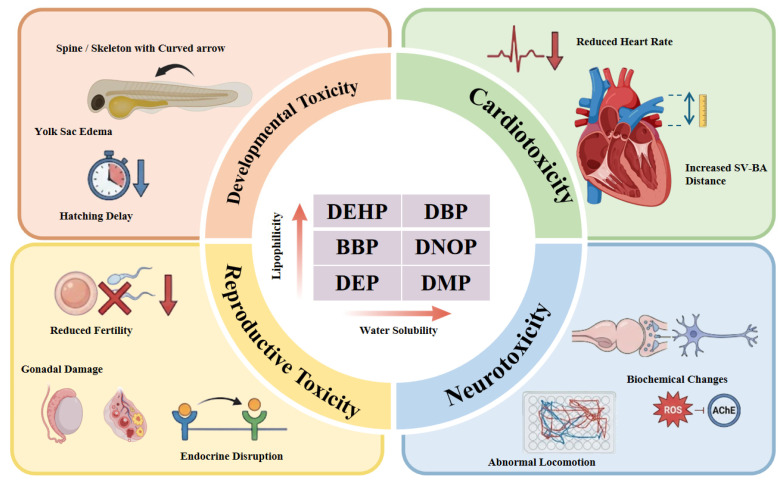
Six priority PAEs, differing in hydrophobicity and bioaccumulation potential, induce multisystem toxicity in zebrafish. Following uptake, PAEs disrupt endocrine signaling, redox homeostasis, and gene regulation, leading to developmental defects, cardiac dysfunction, neurobehavioral impairment, and reproductive toxicity. These effects collectively translate into reduced fitness and potential population-level risks in aquatic organisms.

In this section, we systematically review the phenotypic toxicological endpoints induced by the six representative PAEs across multiple physiological systems in zebrafish. As a highly sensitive biological model, zebrafish respond to PAE exposure through a variety of macroscopic alterations. As summarized in Figure 2 and Table 1, these plasticizers primarily elicit distinct anomalies in developmental, cardiovascular, neurobehavioral, and reproductive systems. The following subsections will dissect these specific systemic impairments in detail, highlighting the most sensitive observable endpoints for each compound and comparing the substantial differences in their descriptive toxicity thresholds.

### 4.1. Developmental Toxicity and Teratogenic Phenotypes in Early Life Stages

Developmental toxicity is one of the most frequently reported toxic effects of PAEs in zebrafish. Due to the rapid embryonic development and optical transparency of zebrafish embryos, developmental endpoints have been widely used to evaluate the toxicity of environmental contaminants. Previous studies have demonstrated that exposure to various PAEs can induce multiple developmental abnormalities in zebrafish embryos and larvae. To assess sublethal effects, developmental and teratogenic toxicity in zebrafish embryos were evaluated, with common abnormalities including altered hatching rate, body size and heart rate, body curvature, yolk sac and pericardial edema, inhibited spontaneous movement, swim bladder malformation, and pigmentation changes [30,40].

Comparative studies have demonstrated pronounced differences in toxicity potency among individual PAEs. For example, a systematic evaluation of six priority PAEs (DMP, DEP, DBP, BBP, DEHP, and DNOP) revealed a clear toxicity ranking based on mortality and developmental defects: DBP > BBP > DEP > DNOP > DEHP > DMP. Among them, DBP exhibited the highest developmental toxicity among the tested PAEs, inducing spinal curvature and pericardial edema at concentrations around 0.5 mg/L, with malformation severity increasing in a concentration-dependent manner. BBP and DEP also showed strong inhibitory effects on embryonic movement and heart rate in a concentration-dependent manner, further supporting their high developmental toxicity. In contrast, DEHP and DNOP generally showed weaker morphological toxicity, particularly at environmentally relevant concentrations.

Although DEHP causes comparatively weaker acute developmental and morphological toxicity than DBP and BBP, it exerts pronounced chronic reproductive and endocrine-disrupting effects at environmentally relevant concentrations. DEHP exposure has been reported to increase deformity rates (up to 20% at 10 mg/L), reduce hatching success even at low concentrations (0.5–2.5 µg/L), and inhibit growth as indicated by decreased body length. Hyperemia and dark pigmentation were also reported in DEHP-exposed embryos [41].

In addition, typical malformations such as spinal curvature, swim bladder defects, and yolk-sac edema have been consistently observed. Importantly, the sensitivity to DEHP appears to be stage-dependent, with early embryonic exposure (e.g., 3–24 hpf) causing more pronounced developmental impairments than later exposure windows. Moreover, prolonged exposure may exacerbate edema-related phenotypes. At the molecular level, DEHP has been shown to upregulate skeletal development-related genes such as *runx2b* (1.44-fold) and *shha*, suggesting that disruption of developmental signaling pathways, including potential interactions with thyroid hormone signaling, may underlie its toxicity.

Overall, PAEs exhibit multi-endpoint developmental toxicity in zebrafish, with both compound-specific and stage-dependent effects. Teratogenic abnormalities, particularly spinal deformities and edema, represent the most sensitive and consistently observed endpoints, especially for high-toxicity compounds such as DBP and BBP. In contrast, compounds with lower apparent morphological toxicity, such as DEHP, may exert subtler but biologically significant effects at the molecular and endocrine levels. These findings highlight that developmental toxicity assessment of PAEs should integrate both phenotypic and mechanistic endpoints to fully capture their potential risks.

### 4.2. Cardiovascular Toxicity and Cardiac Morphogenetic Defects

The cardiovascular system is another important target of phthalate exposure in zebrafish embryos. Because zebrafish embryos develop rapidly ex utero and remain optically transparent during early organogenesis, they have become a widely used in vivo model for cardiotoxicity evaluation [42]. Across the available PAE studies, the most common cardiac phenotypes include reduced or abnormal heart rate, pericardial edema, increased sinus venosus–bulbus arteriosus (SV-BA) distance, defective cardiac looping, and dysregulation of key cardiac developmental regulators, indicating that PAEs can interfere with both cardiac morphogenesis and cardiac function during early development.

Among the individual compounds, DBP and BBP show the clearest and strongest evidence of cardiotoxicity. Sun and Li exposed zebrafish embryos from 4 to 72 hpf to 0, 0.36, 1.8, and 3.6 μM DBP, and found that 1.8 μM already significantly impaired growth, increased overall malformation and cardiac malformation rates, and disrupted cardiac looping, whereas 3.6 μM significantly affected all measured endpoints; *nkx*2.5 and *tbx*5 were also downregulated in a dose-dependent manner [43]. Similarly, Sun and Liu reported that exposure to 0, 0.1, 0.6, and 1.2 mg/L BBP from 4 to 72 hpf caused yolk-sac edema, spinal curvature, tail deformity, and cardiac defects, with 0.6 mg/L significantly increasing the cardiac malformation rate and SV-BA distance while reducing heart rate, and 1.2 mg/L affecting nearly all endpoints; *nkx*2.5 and Tbx5 were again downregulated [36]. Evidence for DEHP is also substantial. In a dedicated heart-development study, injection of 0.02 pg DEHP into the yolk sac at the one-cell stage reduced heart rate at 3–4 dpf, induced pericardial edema in 12.6 ± 1.5% of larvae, and caused marked heart-looping disorders with elongated atrioventricular distance, together with dysregulation of several cardiac transcription factors [27]. A newer study exposing embryos to 0.0086–86 mg/L DEHP through 96 hpf further showed altered *tbx20*, *bcl2*, and *il1b* expression and significant changes in arterial pulse and posterior cardinal vein linear velocity, supporting a functional cardiotoxic effect even when gross pericardial enlargement was limited [31]. For DMP, Cao et al. exposed embryos to sublethal concentrations from 4 to 96 hpf and observed pericardial edema, increased SV-BA distance, and decreased heart rate, stroke volume, ventricular axis shortening rate, and ejection fraction, together with oxidative stress, apoptosis, and disruption of MAPK and calcium signaling pathways [26]. In addition, a head-to-head study of six priority PAEs showed that all tested compounds, including DMP, DEP, DBP, DEHP, DNOP, and BBP, decreased heart rate and induced pericardial edema in zebrafish larvae; importantly, DBP and BBP produced mortality even at low doses, whereas DEHP and DNOP showed comparatively minor morphological effects [25].

Collectively, current toxicological evidence suggests that among the phthalic acid esters (PAEs) congeners investigated, dibutyl phthalate (DBP) exhibits the highest cardiotoxic potential, followed by benzyl butyl phthalate (BBP). Although a parallel comparative study encompassing six PAEs reported a marginally different ranking for general developmental toxicity (e.g., mortality and malformation rates) in the order of DBP > BBP > DEP > DNOP > DEHP > DMP, this disparity further underscores the distinction between organ-specific assessments, such as cardiotoxicity, and holistic developmental evaluations. While the relative toxicological rankings of DEHP, DMP, DEP, and DNOP require further validation through specialized research, the leading cardiotoxicity of DBP and BBP represents the most robust consensus across the current literature [25].

### 4.3. Neurobehavioral Toxicity and Neurodevelopmental Impairment

The nervous system is another major target of phthalate exposure in zebrafish. Because zebrafish embryos and larvae develop externally, remain transparent during early development, and exhibit quantifiable neurobehavioral endpoints such as spontaneous tail coiling, light–dark locomotor activity, and fluorescent neuronal reporter signals, they are widely used for developmental neurotoxicity assessment. Current evidence indicates that phthalic acid esters (PAEs) can impair neurogenesis, disturb neurotransmitter signaling, inhibit acetylcholinesterase activity, and induce oxidative and apoptotic injury in the zebrafish nervous system, suggesting that neurotoxicity is a major component of PAE developmental toxicity. A direct head-to-head study by Tran et al. exposed zebrafish embryos from 2–4 hpf to 120 hpf to 0, 0.5, 5, 50, 500, 1000, and 10,000 μg/L of DMP, DEP, BBP (BBzP), DEHP, and DNOP. In the same assay, DMP, DEP, and DOP did not induce significant locomotor alterations, whereas BBP produced the strongest neurobehavioral response, with significant increase in swimming, bursting, and freezing during the dark phase at 0.5 and 5 μg/L, and a significant reduction in activity during the light phase at all tested concentrations except 1000 μg/L. Mechanistically, BBP at 500–10,000 μg/L reduced Tg(HuC:eGFP) and Tg(mbp:GFP) fluorescence and suppressed the expression, while DEHP reduced fluorescence only in Tg(HuC:eGFP), indicating that BBP is the most neurotoxic compound in this panel [44]. Dedicated studies also show strong neurotoxicity for DBP. Paquette et al. reported that 2.5 μM DBP exposure from the pre-gastrulation stage to 72 hpf disrupted hindbrain structure and rhombomere patterning and induced defects in branchiomotor neuron patterning, migration, and Mauthner neuron development [45]. In adult zebrafish, 28-day exposure to 0.08, 0.4, and 2 mg/L DBP significantly increased MDA and 8-OHdG in brain tissue, with AChE inhibition rates of 13.4%, 11.9%, and 14.7%, respectively, and integrated biomarker response values of 4.37, 7.18, and 9.63 [46]. BBP-specific studies further support its high potency: short-term exposure to 0.332, 0.665, and 1.33 mg/L for 7 days caused concentration-dependent AChE inhibition, with significant inhibition already at 0.665 mg/L, and shifted SOD from induction at 0.332 mg/L to inhibition at 0.665–1.33 mg/L [47]. A later 28-day adult study at 0, 5, 50, and 500 μg/L BBP further demonstrated increased ROS and 8-OHdG, brain apoptosis and histopathological damage, and transcriptomic remodeling with 293 induced and 511 repressed genes [37]. DEHP also showed robust neurobehavioral toxicity: embryos exposed from about 6 hpf for 7 days to 1, 2.5, 5, and 10 mg/L displayed inhibited spontaneous tail movement, reduced locomotor activity, increased apoptosis and MDA, decreased SOD and dopamine, and disturbed expression of *th*, *dat*, *mao*, dopamine receptor genes, and apoptosis-related genes [48]. Although weaker than BBP, DBP, and DEHP, DEP and DMP were not neuroinactive. Xu et al. showed that 500 μg/L DEP significantly inhibited AChE and upregulated *gap43*, elavl3, *gfap*, *mbp*, *α1-tubulin*, and *ngn1* in embryos exposed from 4 to 96 hpf [38]. Yang et al. later found that DMP at 5–100 mg/L from 4 to 120 hpf caused a lower survival rate at 50 mg/L (72.78–78.33% during 24–96 hpf), reduced hatching at 48 hpf to 39.44% and 2.22% at 25 and 50 mg/L, increased spontaneous tail coiling at 24 hpf, decreased swimming distance at 120 hpf, reduced Tg(*elavl3*:EGFP) fluorescence at 50 mg/L, and downregulated *gap43*, mbp, α1-tubulin, and *syn2a* [39].

Collectively, current toxicological research identifies BBP as the most potent neurotoxicant among the six investigated PAE congeners, followed closely by DBP. BBP consistently demonstrates the highest neurotoxic potency; conversely, DNOP was reported to elicit no significant alterations in locomotor activity within the 0.5–10,000 μg/L range. Secondly, DBP has been repeatedly observed to induce biochemical impairments in specialized brain regions, particularly the hindbrain and the adult brain. Thirdly, while DEHP persistently triggers behavioral, dopaminergic, oxidative, and apoptotic changes, these effects typically require higher threshold concentrations than those required for BBP or DBP. Furthermore, while both DEP and DMP produce measurable neurotoxic signatures, the evidence for DEP is primarily centered on acetylcholinesterase (AChE) inhibition and the dysregulation of neurodevelopment-related genes, whereas the effects of DMP are largely restricted to significantly higher doses. Currently, DNOP appears to possess the lowest neurotoxic potential among these compounds. Recent comparative investigations of zebrafish brain tissues further suggest that DEHP induces significantly more pronounced oxidative stress and neurotransmitter imbalances than DNOP [33].

Taken together, the currently available evidence suggests that oxidative stress may represent a central upstream mechanism underlying PAE-induced neurotoxicity in zebrafish. Increased ROS production and antioxidant imbalance appear to trigger neuronal apoptosis and disrupt neurotransmitter homeostasis, particularly dopamine signaling pathways. Similar transcriptome-based evidence has also highlighted the critical involvement of oxidative stress- and apoptosis-related signaling pathways in pollutant-induced neuronal injury models [49,50]. In parallel, inhibition of acetylcholinesterase activity may further impair synaptic transmission and locomotor behavior. The consistency of these findings across multiple studies indicates that oxidative stress, neurotransmitter dysregulation, and apoptosis are mechanistically interconnected rather than independent neurotoxic events. Notably, BBP and DBP appear to induce these neurotoxic responses at substantially lower concentrations than DEP and DMP, supporting a structure-dependent toxicity pattern associated with hydrophobicity and bioaccumulation potential.

### 4.4. Reproductive Toxicity and Endocrine-Disrupting Effects

PAEs are quintessential endocrine-disrupting chemicals that profoundly interfere with the endocrine homeostasis and reproductive health of zebrafish. This systemic disruption is a critical toxicological endpoint, as it directly compromises population sustainability and recruitment in aquatic ecosystems. Due to their high fecundity and well-defined gametogenesis, zebrafish serve as a pivotal model for assessing these impacts through diverse endpoints, including spawning and fertilization rates, gonadal histology, the gonadosomatic index (GSI), and the transcriptional regulation of the hypothalamic–pituitary–gonadal (HPG) axis.

Extensive studies have demonstrated that the severity of reproductive impairment in zebrafish varies significantly among different PAE congeners, typically following a structure-dependent pattern. DEHP and DBP are consistently identified as the most potent reproductive toxicants among the representative PAEs: Chronic exposure to DEHP at environmentally relevant concentrations (20–40 μg/L) significantly reduced egg production and impaired reproductive capacity in female zebrafish [32,51]. This decline is associated with impaired oocyte maturation and the ectopic induction of vitellogenin (VTG) in males. Furthermore, DEHP exhibits significant transgenerational toxicity; parental exposure to 40 μg/L DEHP leads to reduced fertilization and survival rates in F1 and F2 offspring [52,53].DBP exposure at 500 μg/L led to nearly 100% reproductive failure, while 100 μg/L reduced sperm motility by ~30%, accompanied by upregulation of *cyp19a1a*, suggesting disruption of the androgen–estrogen balance. BBP and DNOP exhibited moderate reproductive toxicity, with BBP inducing follicular atresia in 20–30% of females at 0.1–1.0 mg/L [54]. Similarly, long-chain DNOP typically requires concentrations of 1–5 mg/L to induce a statistically significant reduction in the gonadosomatic index (GSI) and fertilization success. Short-chain PAEs such as DEP and DMP exhibit the lowest reproductive potency. Significant toxicological effects, such as minor fluctuations in GSI or spawning frequency, are generally only observed at high experimental doses exceeding 10–50 mg/L [55].

In addition to reproductive toxicity, several studies have demonstrated that DEHP can disrupt thyroid hormone homeostasis in zebrafish by altering T3/T4 levels and the expression of deiodinase-related genes, suggesting additional interference with the hypothalamic–pituitary–thyroid (HPT) axis during development. In summary, the reproductive toxicity of PAEs in zebrafish is characterized by a clear structure–activity relationship, where long-chain or branched congeners (e.g., DEHP, DBP) possess lower effect thresholds than their short-chain counterparts (e.g., DMP). These compounds not only induce immediate reproductive decline in the exposed generation but also threaten the developmental potential of subsequent generations through endocrine-mediated mechanisms. Given that the Lowest Observed Effect Concentrations (LOECs) for DEHP and DBP are approaching the levels detected in contaminated aquatic environments, these compounds remain the primary focus for ecological risk assessments.

### 4.5. Comparative Analysis of Toxicological Sensitivity Among Different PAE Structures

The toxicological profiles of PAEs in zebrafish are profoundly influenced by their chemical structures, particularly the length and configuration of the alkyl side chains. A clear structure–activity relationship is observed: as the alkyl chain length and hydrophobicity (logK_ow_) increase, the bioaccumulation potential and subsequent toxicity generally intensify. Consequently, long-chain PAEs such as DEHP (logK_ow_ ≈ 7.5) and DBP (logK_ow_ ≈ 4.5) exhibit significantly higher potency in inducing developmental and reproductive impairments compared to short-chain congeners like DMP and DEP. For instance, while DEHP can trigger endocrine disruption at microgram-per-liter levels, DMP often requires concentrations in the high milligram-per-liter range to elicit comparable physiological stress [44].

Among the various life stages and physiological systems evaluated, the embryonic developmental system is identified as the most sensitive target for PAE exposure. Zebrafish embryos, characterized by rapid organogenesis and high metabolic activity, exhibit lower tolerance to chemical insults than adults [56]. Comparative studies indicate that morphological endpoints—such as heart rate, hatching success, and teratogenic malformations (e.g., pericardial edema)—respond to PAEs at concentrations significantly lower than those required to cause adult mortality [57].

Toxicological effects in zebrafish typically manifest within an experimental range of 10 μg/L to 20 mg/L. Specifically, the LOECs for potent congeners like DEHP and DBP cluster between 20 μg/L and 1 mg/L, whereas short-chain congeners (DEP and DMP) require significantly higher concentrations (10–100 mg/L) that often exceed industrial effluent levels [44].

However, the environmental relevance of these laboratory-derived thresholds remains a subject of critical inquiry. While high-dose exposures elucidate fundamental molecular mechanisms, they may not reflect the chronic, low-level “cocktail” of contaminants characteristic of natural systems [58].

Overall, the comparative evidence supports a strong relationship between hydrophobicity, bioaccumulation potential, and toxicological potency among PAEs. Long-chain congeners with higher logKow values, such as DEHP and DNOP, exhibit stronger lipid affinity and greater accumulation in zebrafish tissues, particularly during embryonic development. However, DBP and BBP frequently demonstrate lower LOEC values and more pronounced developmental and neurobehavioral toxicity despite having lower hydrophobicity than DEHP, suggesting that toxicity is not determined solely by bioaccumulation potential. Differences in endocrine-disrupting activity, molecular target interactions, and metabolic transformation may additionally contribute to compound-specific toxicity profiles. These observations indicate that both physicochemical properties and biological pathway specificity jointly determine PAE toxicological outcomes in zebrafish.

Methodological Considerations: We note that methodological heterogeneity across the reviewed studies—including variations in exposure duration (e.g., 4–72 hpf vs. 2–120 hpf), exposure protocols (static vs. flow-through systems), concentration ranges, and endpoint selection—introduces inherent limitations to cross-study comparisons. Some discrepancies in reported toxicity thresholds may reflect methodological differences rather than true compound-specific variations. We have explicitly discussed these caveats throughout the review and recommend that future PAE toxicological studies adopt standardized protocols (e.g., OECD TG 236 for fish embryo acute toxicity) to facilitate more robust meta-analyses and regulatory assessments. A comparative summary of the toxicity profiles, including logK_ow values, most sensitive biological systems, typical toxicity endpoints, approximate LOECs, and relative toxicity rankings of the six representative PAEs, is provided in Table 2.

## 5. Environmental Relevance Assessment: Comparative Analysis of Laboratory Toxicity Thresholds and Real-World Exposure Risks

### 5.1. Environmental Occurrence and Spatiotemporal Distribution of Priority PAEs in Aquatic Ecosystems

PAEs have emerged as ubiquitous organic contaminants in global aquatic ecosystems, primarily due to their massive production volumes and their tendency to leach from plastic products, as they are not chemically bonded to the polymer matrix [11,60]. Upon entering the environment through industrial discharge, wastewater treatment plant effluents, and surface runoff, PAEs enter hydrological cycles, where they persist as a chronic threat to aquatic biodiversity. Their presence has been documented in diverse geographical locations, ranging from highly industrialized urban rivers to remote lacustrine environments.

Consequently, PAEs have been frequently detected in various environmental matrices, including surface water, sediments, soils, and biota. Monitoring studies conducted in different regions have reported the presence of several commonly used PAEs, such as DEHP, DBP, BBP, DEP, and DMP, in aquatic environments. The distribution of these congeners is largely dictated by their physicochemical properties; shorter-chain PAEs are more prevalent in the dissolved phase due to their higher water solubility, whereas long-chain, hydrophobic congeners exhibit a strong affinity for suspended particulate matter and often accumulate in sediments at concentrations several orders of magnitude higher than in the overlying water.

### 5.2. Correlation Between Laboratory Toxicity Thresholds and Environmentally Realistic Concentrations

A fundamental challenge in assessing the ecological risks of PAEs lies in the inherent discrepancy between laboratory-controlled exposure and actual environmental conditions. The exposure concentrations typically employed in laboratory toxicity studies differ significantly from the levels detected in natural environments. While high-dose experiments are indispensable for establishing dose–response relationships, comparing measured environmental concentrations with experimental exposure levels is paramount for evaluating the environmental relevance of laboratory-derived toxicity data. This comparison provides the necessary context to determine whether the biological impairments observed in zebrafish are likely to occur under current environmental pressures.

Environmental monitoring studies generally report PAE concentrations in aquatic environments ranging from low ng/L to low μg/L levels. For instance, in relatively “clean” or moderately urbanized river systems, such as the Yangtze River, total PAE concentrations typically range from 0.04 to 2.46 μg/L. In contrast, many zebrafish toxicity studies utilize exposure concentrations ranging from mid-μg/L to mg/L levels to investigate potential toxic effects. Notably, the lowest observed effect concentrations for the reproductive and DEHP and DBP are commonly cited as 20 μg/L and 100 μg/L, respectively. Despite this gap, peak environmental concentrations recorded in industrial hotspots—such as the 345.6 μg/L measured in the Haihe River or the 125.0 μg/L in Northern Vietnam—actually exceed these laboratory toxicity thresholds, indicating an immediate risk to local fish populations.

The implementation of higher concentrations in laboratory studies is often intended to elucidate potential toxic mechanisms or to identify sensitive biological endpoints that may not be apparent at lower doses. High-dose regimes enable researchers to observe acute molecular responses, such as the rapid upregulation of vitellogenin or the manifestation of cardiac edema, within manageable experimental timeframes. However, it is imperative to interpret these findings within the context of environmentally relevant exposure levels. Given that monitoring data indicate the ubiquitous presence of PAEs even at sub-threshold levels, future research must increasingly focus on chronic, low-dose “cocktail” exposures to better reflect the sustained environmental stress encountered by aquatic organisms. These findings indicate that certain environmentally detected PAE concentrations may fall within ecologically relevant effect ranges observed in zebrafish toxicity studies.

### 5.3. From Individual Zebrafish Anomalies to Broader Ecological Shifts

Toxic effects observed at the individual level, including developmental aberrations, endocrine disruption, and metabolic shifts, harbor profound implications for the broader health of aquatic ecosystems. Adverse impacts on early ontogeny, behavioral patterns, and reproductive success in zebrafish do not occur in isolation; rather, they can fundamentally alter the survival, growth, and overall population dynamics of aquatic organisms. Such sub-lethal impairments often result in a gradual decline in population recruitment, which may eventually lead to shifts in community structure and a loss of biodiversity within contaminated habitats. For example, developmental abnormalities such as pericardial edema and spinal curvature, often coupled with behavioral alterations induced by PAE exposure, significantly diminish the ability of zebrafish larvae to evade predators or effectively locate food resources. Studies have demonstrated that exposure to DEHP or DBP can disrupt locomotor activity by altering neurotransmitter levels and downregulating genes related to muscle development, such as *vmhc* and *actc1b*. These physiological handicaps directly translate to increased mortality in the wild, as impaired larvae are unable to perform the rapid “C-start” escape responses necessary to survive predation. Furthermore, the documented reduction in egg production ensures that even surviving individuals contribute less to the subsequent generation, compounding the ecological debt. Therefore, the toxic effects of PAEs observed in zebrafish studies provide valuable insights into the potential ecological consequences of PAE contamination in aquatic environments. By serving as a sensitive sentinel species, zebrafish provide a biological “early warning system” that allows for the prediction of long-term ecological risks that might otherwise remain undetected until large-scale population collapses occur.

### 5.4. Environmental Risk Assessment Based on Zebrafish-Derived Toxicity Thresholds

A widely used ecological risk assessment metric is the Risk Quotient (RQ), calculated as the ratio between the Measured Environmental Concentration (MEC) and the Predicted No-Effect Concentration (PNEC), where RQ = MEC/PNEC. Given the ubiquitous presence and multi-organ toxicity of PAEs, rigorous environmental risk assessment is paramount for prioritizing chemical regulation and mitigating the degradation of aquatic biodiversity. Establishing protective thresholds requires a synthesis of both the chemical occurrence data and the biological sensitivity of model organisms like the zebrafish. Researchers estimate the potential ecological risks posed by PAEs in aquatic ecosystems by comparing toxicity thresholds—such as LOEC, NOEC, or values—with measured environmental concentrations. A widely adopted metric is the Risk Quotient, defined as the ratio of the MEC to the Predicted No-Effect Concentration. In several industrialized river basins, such as the Haihe River in China or urban canals in Southeast Asia, the RQ values calculated for DEHP and DBP frequently exceed 1.0, indicating a high probability of adverse ecological effects on resident fish populations.

To refine these risk considerations, future research must move beyond single-compound assessments and address the mixture toxicity of multiple PAEs and their metabolites. Moreover, a comprehensive risk assessment of these pollutants must account for multigenerational consequences and sub-lethal genomic alterations, rather than focusing solely on the current generation. Such an integrated approach is vital to characterize the protracted toxicological legacy that these recalcitrant plastic additives leave for future aquatic populations.

From a regulatory perspective, the ecological risks associated with PAEs are commonly evaluated using predicted environmental concentration (PEC) and predicted no-effect concentration (PNEC) frameworks. In several contaminated aquatic environments, measured environmental concentrations of DEHP and DBP approach or exceed experimentally derived LOEC values in zebrafish, suggesting potential ecological risks to aquatic organisms. According to commonly applied ecological risk assessment criteria, risk quotient (RQ = PEC/PNEC) values greater than 1 indicate a high ecological concern.

Furthermore, regulatory agencies including the United States Environmental Protection Agency (US EPA) and the European Food Safety Authority (EFSA) have identified several PAEs, particularly DEHP and DBP, as priority pollutants or endocrine-disrupting chemicals requiring environmental monitoring and exposure control. These regulatory perspectives further support the environmental relevance of zebrafish-derived toxicity data.

Importantly, natural aquatic systems are characterized by chronic low-dose co-exposure to multiple contaminants rather than isolated single-compound exposure. Therefore, future ecological risk assessments should increasingly consider mixture toxicity, cumulative endocrine-disrupting effects, and long-term multigenerational impacts under environmentally realistic exposure conditions. Such integrative approaches are essential for improving the ecological relevance and regulatory applicability of zebrafish toxicological studies.

## 6. Limitations

Several limitations should be acknowledged in the present review. First, although extensive literature retrieval and comparative synthesis were conducted, the review was designed as a comparative narrative review rather than a formal systematic review or meta-analysis. Therefore, quantitative pooled effect estimation and formal risk-of-bias scoring were not performed. Second, substantial heterogeneity exists among zebrafish toxicity studies regarding exposure duration, developmental stage, concentration range, and endpoint selection, which may influence direct cross-study comparability. Third, many currently available studies primarily focus on acute single-compound exposure, whereas environmentally realistic chronic low-dose mixture exposure remains insufficiently investigated. Finally, despite increasing evidence regarding molecular and endocrine-disrupting mechanisms, the long-term ecological and multigenerational consequences of PAE exposure in aquatic systems remain incompletely characterized. These limitations highlight important directions for future zebrafish-based ecotoxicological research.

## 7. Conclusions and Future Perspectives

In summary, the toxicological impacts of PAEs on zebrafish models are highly dependent on their chemical structures, presenting clear descriptive differences in toxicity. Long-chain and branched congeners, such as DEHP and DBP, exhibit significantly higher toxicity, inducing severe developmental defects and reproductive decline at concentrations as low as 0.5–20 μg/L. In contrast, short-chain congeners like DMP and DEP elicit phenotypic abnormalities only at much higher laboratory doses. Early embryonic stages, as well as the cardiovascular, neurobehavioral, and reproductive systems, represent the most sensitive biological targets. This is supported by the findings in Section 4.3, where BBP and DBP exhibited significant neurobehavioral toxicity comparable to their cardiovascular effects. Crucially, comparing these observable toxicological endpoints with environmental monitoring data reveals that real-world PAE concentrations in industrial hotspots frequently reach or exceed the LOECs. This overlap underscores that PAE contamination poses a direct and pressing threat to the survival and population dynamics of aquatic life.

Moving forward, ecological risk assessments should bridge the gap between acute laboratory observations and actual environmental conditions. Future research should shift away from single-compound, high-dose exposures and prioritize evaluating the chronic, low-dose impacts of PAEs across the entire zebrafish life cycle. Additionally, since PAEs exist as complex mixtures in natural water bodies, investigating their joint toxicity alongside other common aquatic contaminants is essential for accurate risk profiling. Finally, considering the persistent nature of reproductive impairments, further observational studies on the transgenerational toxicity of PAEs are warranted to fully characterize their long-term ecological consequences and legacy effects on future aquatic generations.

## Figures and Tables

**Table 1:** Typical Toxicity Profiles of Representative PAEs in Zebrafish.

Compound	Toxicity Type	Life Stage	Zebrafish Strains	Exposure Duration	LOEC (mg/L)	Concentration Range Tested (mg/L)	Quantified Phenotypes	Molecular/Biochemical Markers	Endocrine Axis	Most Sensitive System	References
DEHP	Developmental	0–96 hpf	Wild-type	6–120 hpf	0.0005	1, 5, 10, 20	Hatching delay, spinal curvature	*sox9a*, *col2a1 ↓*	–	Skeletal system	[30]
	Cardiotoxicity	6–72 hpf	Wild-type	6–72 hpf	0.25	1, 5, 10, 20	Pericardial edema, heart rate ↓	*myl7*, *tbx5 ↓*	–	Cardiovascular system	[31]
	Reproductive	Adult	Wild-type	30–90 dpf	0.02–0.04	1, 10, 100	Gonadal dysplasia, fertility ↓	–	HPG	Reproductive system	[32]
	Thyroid toxicity	Larva	Wild-type	120 hpf	1	1, 10, 100	T3/T4 levels altered	deiodinase genes	HPT	Endocrine system	[26]
	Neurotoxicity	Embryo/Larva	Wild-type	0–96 hpf	–	0.1–1.0 (typical testing range in PAE studies)	Oxidative stress, neurotransmitter disorder	*AChE ↓*, *SOD ↓*, *CAT ↓*	–	Nervous system	[33]
DBP	Developmental	Embryo	AB	24–96 hpf	0.5	0.1, 0.5, 2.5, 12.5	Yolk-sac retention, tail malformation	*raldh2*	*–*	Digestive/Whole body	[25]
	Reproductive	Adult	AB	60–90 dpf	0.1	0.1, 1, 10	Fecundity ↓	*vtg1*	HPG	Reproductive system	[25]
	Spinal toxicity	Embryo	Wild-type	0–96 hpf	–	0.01, 0.1, 1	Spinal defects, motor abnormalities	Key genes of notochord, muscle, bone altered	–	Skeletal/Motor system	[34]
	Integrated toxicity	Embryo → Juvenile/Adult	Wild-type	Subchronic	0.0438 mg/L (reproductive toxicity)	0, 0.0049, 0.0136, 0.0438	Severe oxidative stress, neurotoxicity, hepatic DNA damage	↑ ROS, ↑ 8-OHdG (DNA oxidation), ↓ AChE, altered HPG/HPT genes	HPG/HPT axis disruption	Multiple systems	[35]
BBP	Developmental	Embryo	Wild-type	4–72 hpf	0.6 mg/L	0, 0.6, 1.2	Developmental delay, skeletal malformation	*Nkx*2.5 ↓, *Tbx*5 ↓	Developmental	Cardiovascular system	[36]
	Neurotoxicity	Adult embryo/larval	Wild-type	–	Not specified	–	Oxidative stress, apoptosis malformation	Brain transcriptome changes	–	Neurological	[37]
DNOP	Neurotoxicity	Embryo/Larva	Wild-type	0–96 hpf	–	0.1–1 (as tested in PAE mixture)	Oxidative stress, neurotransmitter disorder (weaker than DEHP)	Suppression of cell cycle and DNA replication genes (transcriptomic changes)	–	Nervous system	[33]
DEP	Neurotoxicity	7–28 dpf	AB	7–28 dpf	50	50, 100, 200	Spontaneous locomotion ↓	–	–	Nervous system	[38]
	Endocrine	Adult	Wild-type	30 d	0.1	0.1–1	Plasma E2 ↓	*esr1 ↓*	HPG	Endocrine system	–
DMP	Developmental	Embryo	Wild-type	2–144 hpf	>10	10, 50, 100, 200	Lethality, malformation at high doses	–	–	Whole body	[39]

–: Not reported or not applicable. ↓ indicates a decrease or reduction.

**Table 2:** Comparative toxicity profiles of representative PAEs in zebrafish.

Compound	logK_ow_	Most Sensitive System	Typical Toxicity Endpoints	Approximate LOEC (mg/L)	Relative Toxicity	References
DBP	4.5–5.1	Cardiovascular/Neurobehavioral/Reproductive	Heart rate ↓, fecundity ↓, spinal malformation	0.1–0.5	High	[25,34,59]
BBP	4.7–5.2	Cardiovascular/Neurotoxicity	Skeletal malformation, neurobehavioral defects	0.01–0.5	High	[1,11]
DEHP	7.3–8.4	Reproductive/Endocrine	Hatching delay, thyroid disruption, cardiotoxicity	0.02–1	Moderate–High	[3,10]
DNOP	8.9	Neurotoxicity	Neurotoxicity (weaker than DEHP)	–	Moderate	[10]
DEP	2.4–2.8	Developmental	Pericardial edema, spinal curvature	0.1–0.5	Moderate	[11]
DMP	1.6–2.0	Developmental	Hatching delay, body length ↓	>10	Low	[6]

Note: ↓ indicates a decrease or reduction (e.g., heart rate ↓ = decreased heart rate).

## Data Availability

Data sharing is not applicable to this article as no new data were created or analyzed.
